# Scarlet Fever in England

**Published:** 1864-01

**Authors:** 


*Scarlet Fever in England.*—London is at present suffering from a more formidable epidemic of scarlet fever than has prevailed there for upwards of twenty years, and the disease is rife all over England. Of the total number of deaths in London during the first eight months of the past year, one in every fourteen was due to scarlatina.

